# Intracellular Cholesterol Trafficking and Impact in Neurodegeneration

**DOI:** 10.3389/fnmol.2017.00382

**Published:** 2017-11-17

**Authors:** Fabian Arenas, Carmen Garcia-Ruiz, Jose C. Fernandez-Checa

**Affiliations:** ^1^Department of Cell Death and Proliferation, Instituto de Investigaciones Biomédicas de Barcelona, Consejo Superior de Investigaciones Científicas, Barcelona, Spain; ^2^Liver Unit and Hospital Clinic I Provincial, IDIBAPS, Barcelona, Spain; ^3^Centro de Investigación Biomédica en Red, CIBEREHD, Barcelona, Spain; ^4^Southern California Research Center for ALDP and Cirrhosis, Los Angeles, CA, United States

**Keywords:** cholesterol, lysosomes, mitochondria, endoplasmic reticulum, mitochondria associated ER membranes, Alzheimer disease, Parkinson disease, Niemann Pick type C disease

## Abstract

Cholesterol is a critical component of membrane bilayers where it plays key structural and functional roles by regulating the activity of diverse signaling platforms and pathways. Particularly enriched in brain, cholesterol homeostasis in this organ is singular with respect to other tissues and exhibits a heterogeneous regulation in distinct brain cell populations. Due to the key role of cholesterol in brain physiology and function, alterations in cholesterol homeostasis and levels have been linked to brain diseases and neurodegeneration. In the case of Alzheimer disease (AD), however, this association remains unclear with evidence indicating that either increased or decreased total brain cholesterol levels contribute to this major neurodegenerative disease. Here, rather than analyzing the role of total cholesterol levels in neurodegeneration, we focus on the contribution of intracellular cholesterol pools, particularly in endolysosomes and mitochondria through its trafficking via specialized membrane domains delineated by the contacts between endoplasmic reticulum and mitochondria, in the onset of prevalent neurodegenerative diseases such as AD, Parkinson disease, and Huntington disease as well as in lysosomal disorders like Niemann-Pick type C disease. We dissect molecular events associated with intracellular cholesterol accumulation, especially in mitochondria, an event that results in impaired mitochondrial antioxidant defense and function. A better understanding of the mechanisms involved in the distribution of cholesterol in intracellular compartments may shed light on the role of cholesterol homeostasis disruption in neurodegeneration and may pave the way for specific intervention opportunities.

## Introduction

As amphipathic sterol, cholesterol exerts key structural and physiological functions in all cells. Its planar and rigid structure regulates the fluidity and permeability of the phospholipid bilayer and for the solutes and ions, respectively (Segatto et al., 2014). Moreover, the functions of membrane proteins are modulated by cholesterol, which also participates in many membrane trafficking and transmembrane signaling processes (Egawa et al., 2015). Accordingly, the plasma membrane (PM) of all eukaryotic cells have the cholesterol as both structural and functional essential component, since also works as a precursor for the biosynthesis of bile acids, steroid hormones, and vitamin D which, in turn, have important biological roles as signal transducers and lipid solubilizers (van der Wulp et al., 2013).

Outside the central nervous system (CNS), cellular cholesterol requirement is assured by its *de novo* synthesis and intracellular uptake from lipoprotein-containing particles derived from the diet. However, because the blood-brain barrier (BBB) is impermeable to the plasma lipoproteins, nearly all cholesterol existing in the brain is produced by *de novo* synthesis (Zhang and Liu, 2015). In addition, the degradation and excretion of cholesterol from the brain is mainly driven by the neuron-specific cytochrome P450 oxidase CYP46A1, which hydroxylates cholesterol to 24S-hydroxycholesterol (24-OHC), the most abundant oxysterol in the brain (Gamba et al., 2015), that unlike cholesterol, can cross the BBB, entering the circulation for its disposal by the liver.

Although, the brain makes up only 2.1% of body weight, it contains 23% of the total body cholesterol and exhibits a 10-fold higher cholesterol concentration compared to other tissues (Vance, 2012). Most (at least 99%) of the brain cholesterol is present in an unesterified form, which is distributed in two major pools: (i) Close to 70% is present in the myelin sheaths of oligodendrocytes (white matter); and (ii) and the 30% remainder is present within the plasmalemmal and subcellular membranes of astrocytes and neurons (gray matter). Across the different cell types of the CNS *de novo* synthesis of cholesterol is not homogenous. For instance, oligodendrocytes (responsible for axon myelinization) have a higher capacity for cholesterol biosynthesis than astrocytes, which in turn, exhibit at least 2- to 3-fold higher capacity than neurons (van der Wulp et al., 2013; Segatto et al., 2014; Egawa et al., 2015). During the perinatal period and adolescence the neurons needs encircled in myelin and consequently is when most of brain cholesterol accumulates. After myelination, the whole bulk of cholesterol in the adult brain is maintained at a very low levels with minimal loss (half-life up to 5 years), although it has been suggested that the turnover rate could vary between the different cell types of the CNS, being very high in some neurons with turnover estimates of about 20% per day (Moutinho et al., 2016). Besides the cell-specific rate of brain cholesterol synthesis and turnover, there is evidence indicating a region-dependence of cholesterol synthesis, which is influenced by aging and gender (Segatto et al., 2014). Thus, alterations in the exquisite feedback mechanisms that regulate brain cholesterol homeostasis could be either the cause or the consequence of a number of neurodegenerative disorders.

As the contribution of the absolute levels of cholesterol in major prevalent neurodegenerative diseases, such as Alzheimer disease (AD), is controversial, as discussed below, in this review we focus on the putative contribution of intracellular cholesterol pools, particularly in the endolysosomes and mitochondria, in the development of neurodegeneration. A further understanding of the mechanisms and functional consequences associated with the disruption in the intracellular cholesterol trafficking and accumulation may provide novel opportunities for the treatment of major neurological diseases.

## Cholesterol metabolism in the CNS

### Biosynthesis, homeostasis, and turnover

**Figure 1:**
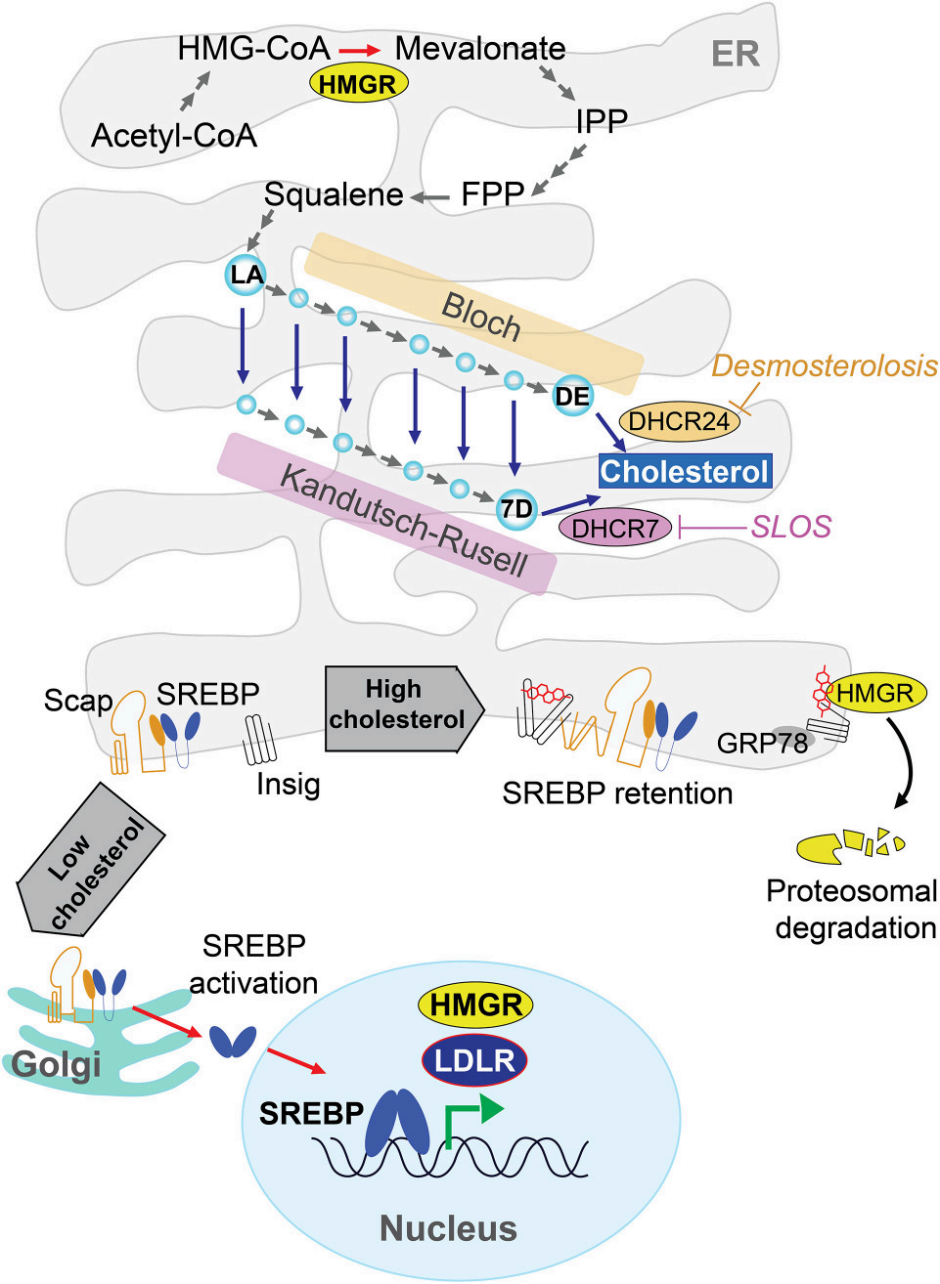
*De novo* cholesterol synthesis in the brain. Cells synthesize cholesterol at endoplasmic reticulum (ER) from acetyl-CoA through several energy demanding enzymatic steps. The rate-limiting enzyme 3-hydroxy-3-methylglutaryl-CoA (HMG-CoA) reductase (HMGR) converts irreversibly HMG-CoA to mevalonate, followed by multi-step enzymatic reactions until lanosterol (LA) and finally to cholesterol by 19-step process involving the Bloch and Kandutsch-Russel pathways. These pathways use desmosterol (DE) or 7-dehydroxycholesterol (7D) as precursors of cholesterol through DHCR24 and DHCR7, respectively. Mutations in these genes cause rare hereditary neurological disease such as Desmosterolosis or Smith-Lemli-Opitz syndrome (SLOS). The cholesterol biosynthesis has a sterol-dependent feedback control that modulates the HMGR levels. ER-sterol accumulation triggers both proteasome-mediated degradation of HMGR through an Insig/GRP78-dependent mechanism and the ER-retention of sterol regulatory element protein 2 (SREBP2), which in turn is controlled by sterol-sensitive SREBP cleavage activating protein (SCAP).

Although, all mammalian cells have the capacity to biosynthesize cholesterol *de novo*, this process is crucial for the CNS since cholesterol's metabolism in the brain occurs isolated to the rest of the organism due to the BBB. Cellular cholesterol is synthesized primarily in the endoplasmic reticulum (ER) from acetate in a complex and resource-intense process that involves over 30 enzymatic steps, initiated by the conversion of acetyl-CoA to 3-hydroxy-3-methylglutaryl-CoA (HMG-CoA) and then irreversibly converted to mevalonate by the rate-limiting enzyme, HMG-CoA reductase (HMGR). This is followed by multi-step enzymatic reactions that sequentially convert mevalonate into 3-isopenenyl pyrophosphate, farnesyl pyrophosphate, squalene, lanosterol, and finally to cholesterol in a 19-step process, involving two distinct but related pathways (Figure 1). The Bloch and Kandutsch-Russel pathways use desmosterol and 7-hydroxycholesterol as the immediate precursors of cholesterol through their reduction by 24-dehydrocholesterol reductase (DHCR24) and 7-dehydrocholesterol reductase (DHCR7), respectively (Martin et al., 2014). Mutations in the genes encoding DHCR24 and DHCR7 cause rare hereditary neurological diseases, illustrating the importance of cholesterol homeostasis in brain physiology (Figure 1, see below). Unlike axons, *de novo* synthesis of cholesterol occurs in neuronal somata of adult neurons, which have minor capability than astrocytes to produce cholesterol. Thus, in neurons the newly synthesized cholesterol needed an anterograde soma-to-axon transport (Zhang and Liu, 2015).

The cholesterol biosynthesis has a crucial feedback control that involves sterol-mediated degradation of HMGR (Figure 1). Accumulation of sterols in the ER membranes triggers the ubiquitination of HMGR by Insig-1 and Insig-2 and the subsequent proteasome-mediated degradation of HMGR. In addition, HMGR gene expression is regulated by ER-bound membrane transcription factor sterol regulatory element protein 2 (SREBP2), whose activation depends on the sterol-sensitive SREBP cleavage activating protein (SCAP). The ER-sterol containing is sensed by SCAP to retain full-length SREBP2 at the ER. Upon ER-sterol depletion, SCAP interacts with SREBP2 to exit the ER and move to the Golgi apparatus, where SREBP2 is sequentially cleaved by the specific proteases S1P and S2P. This event releases the soluble N terminus domain of SREBP2, which travels to the nucleus to induce the transcription of HMGR as well as low-density lipoprotein receptor (LDLR; Garcia-Ruiz et al., 2009; Martin et al., 2014; Fernández-Checa, 2015).

**Figure 2:**
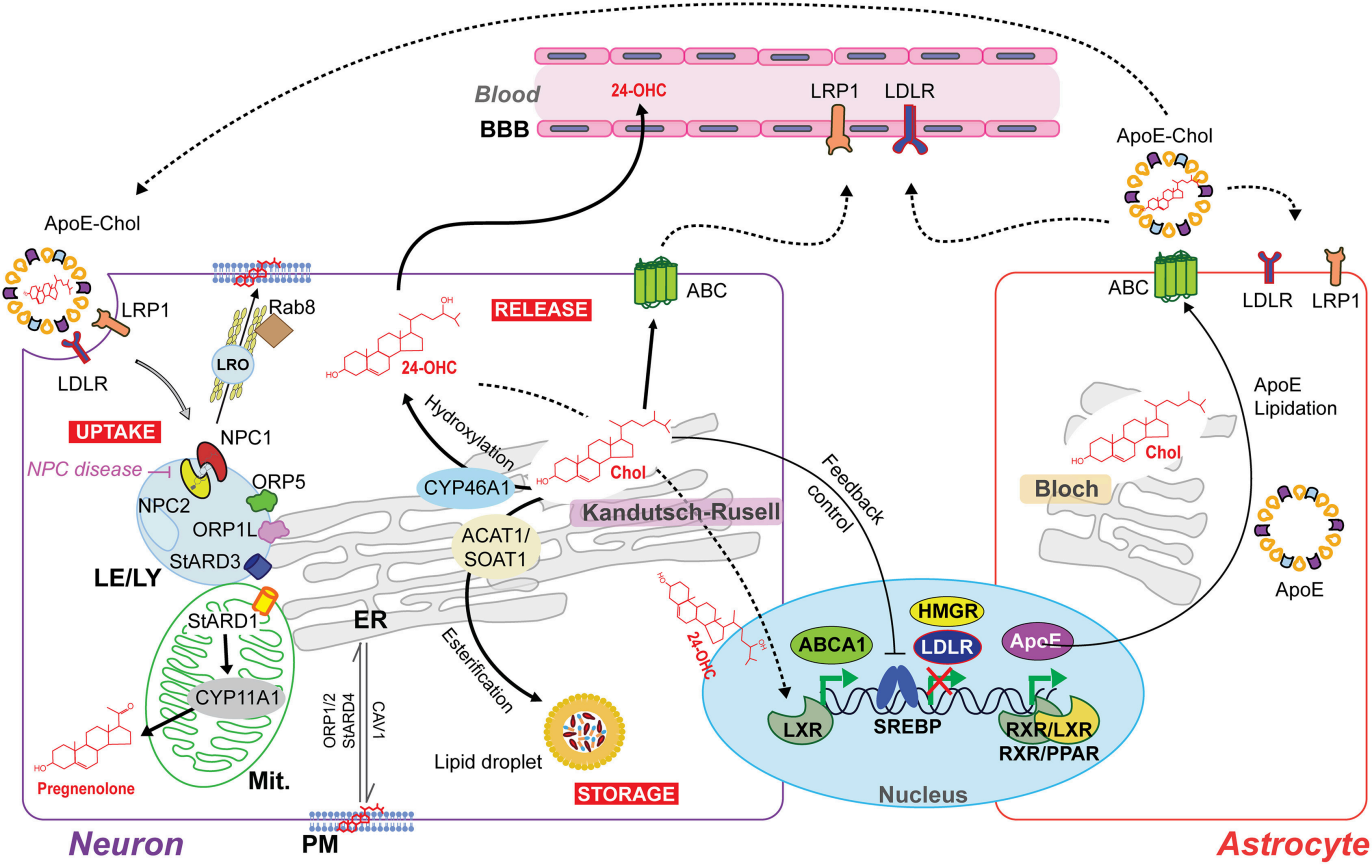
Intercellular and intracellular cholesterol homeostasis in the brain. The Bloch and Kandutsch–Russell pathways involved in cholesterol synthesis operate predominantly in astrocytes and neurons, respectively. The cholesterol produced by astrocytes is delivered to neurons by the secretion of cholesterol-rich apolipoprotein E (ApoE-Chol). Cholesterol is taken by the cells through a receptor-mediated endocytosis of ApoE-Chol, followed by its hydrolysis within late endosome/lysosome (LE/LY) generates free cholesterol. Niemann-Pick type C1 (NPC1) and C2 (NPC2) mediate cooperatively the exit of cholesterol out of LE/LY to reach at subcellular membrane compartments. Defects in NPC1/2 cause the lysosomal storage disorder NPC disease. The intracellular redistribution of cholesterol occurs by vesicular and non-vesicular transport. NPC1 may induce the formation of a cholesterol-enriched lysosome-related organelle (LRO) to cholesterol's delivery at plasma membrane (PM) by a Rab8-dependent movement along actin filaments. Non-vesicular cholesterol transport occurs at intra-organelles membrane contact sites and involves several oxysterol binding proteins (ORP) and steroidogenic acute regulatory proteins (StAR). ORP1L, ORP5 and STARD3 are involved in LE/LY-to-ER transferring cholesterol. STARD1 governs the delivery of cholesterol from the outer mitochondrial membrane (OMM) to the inner mitochondrial membrane (IMM) to generate pregnenolone (neurosteroids precursor) by cytochrome p450scc (CYP11A1). Cholesterol overload is handle through (i) its esterification by acylcoenzyme A:cholesterol acyltransferase 1 (ACAT1/SOAT1) and subsequent intracellular storage in lipid droplets; and through (ii) release via ATP-binding cassette (ABC) transporters or after CYP46A1-dependent conversion to the oxysterol 24S-hydroxycholesterol (24-OHC), which, unlike non-oxydized cholesterol, can freely cross the blood-brain barrier (BBB) and upregulate ABCA1 expression via activation of nuclear liver X receptor (LXR). Moreover, LXR can form heterodimers with retinoid X receptor (RXR) to upregulate ApoE transcription.

The cellular machinery that responds to an excess of cholesterol is expressed at higher levels in neurons than astrocytes. Thus, neurons may handle cholesterol overload by different mechanisms, involving cholesterol esterification and the concomitant intracellular storage in lipid droplets, direct excretion through ATP-binding cassette (ABC) transporters or conversion to 24-OHC through CYP46A1 (Figure 2). The esterification of cholesterol is catalyzed by acylcoenzyme A:cholesterol acyltransferase 1 (ACAT1/SOAT1) in the ER (Zhang and Liu, 2015) and the accumulation of cholesterol esters in lipid droplets represents up to 1% of the total cholesterol content in neurons. However, in conditions where cholesterol homeostasis is disrupted due for instance to the lack of ApoE or cholesterol overload, ACAT1/SOAT1 becomes active in astrocytes (Karten et al., 2006). In addition, it has been observed in *Drosophila* that the accumulation of lipid droplets in glia is a consequence of reactive oxygen species (ROS) and mitochondrial defects (Liu et al., 2015). These findings imply that glial lipid droplets accumulation related with mitochondrial defects in neurons could be an evolutionarily conserved process, emerging as a biomarker for neurodegenerative disease at early stages (Liu et al., 2015).

Neurons express proteins of the ABC subfamily A transporters (ABCA) to control the cellular cholesterol efflux at PM's level (Figure 2). This pathway for cholesterol elimination from neurons is independent of CYP46A1. Compared to astrocytes, the higher expression of ABCA1 in neurons mediates the cholesterol efflux to both lipid-free apolipoprotein A-I (ApoA-I) and E (ApoE) present in cerebrospinal fluid (CSF). These lipoproteins, in turn, can be removed from the brain through lipoprotein receptor-related protein 1 (LRP1; Koldamova et al., 2014). Recently, it has been described that LRP1 is crucial to limit intracellular cholesterol accumulation through inhibition of cholesterol biosynthesis via TGFβ-induced enhanced Wnt5a expression, increasing cholesterol export by stimulation of ABCA1 expression (El Asmar et al., 2016). Interestingly, the Wnt signaling pathway prevents mitochondrial dysfunction in neurons and protects from neurodegeneration (Arrazola et al., 2015).

On the other hand, the hydroxylation of cholesterol to 24-OHC by CYP46A1 constitutes the major excretion pathway of cholesterol in the brain (40–50% of brain cholesterol turnover) since unlike non-oxidized cholesterol, 24-OHC can cross BBB at very higher rate than cholesterol itself (Figure 2). Interestingly, the induction of the activation of nuclear liver X receptor (LXR) by 24-OHC can upregulate ABCA1's expression, which can influence whole-brain cholesterol homeostasis (Zhang and Liu, 2015). Restricted to somata and dendrites (not in axons or presynaptic terminals) of pyramidal cells of the cortex and Purkinje cells of cerebellum (almost undetectable in astrocytes), the expression of CYP46A1 is located mainly in the ER (Moutinho et al., 2016). Additionally, both the plasma and CSF levels of 24-OHC are altered in several neurodegenerative disorders, such as AD, multiple sclerosis (MS), Parkinson disease (PD), and Huntington disease (HD; Moutinho et al., 2016).

### Cholesterol transport

The requirement of neurons for cholesterol is very high, as it is used in neurite formation, maintenance, and synaptic connectivity. Adult neurons meet their cholesterol needs from the generation of cholesterol from astrocytes, which are able to secrete cholesterol-rich lipoproteins containing ApoE (the main lipid transporter in the CNS) that are taken by neurons via endocytosis mediated by LDLR and LRP1 (Figure 2). Notably, the expression of LDLR family is particularly enriched in neurons in specific brain subcellular sites. For instance, multiple cell types of the BBB (e.g., pericytes, neurons, and astrocytes) express LRP-1 principally at the abluminal side (Zlokovic, 2010), where it is able to recognize and participate in the endocytosis of several ligands including ApoE, amyloid precursor protein (APP), and amyloid-β peptide (Aβ; Ramanathan et al., 2015).

On the other hand, chronic high levels of total serum cholesterol increase the brain expression and secretion of ApoE from glial cells (Petanceska et al., 2003). Among the three major human isoforms of ApoE, ApoE3 is most representative isoform (78%), while ApoE4 (15%) and ApoE2 (8%) isoforms are less representatives in humans (El Gaamouch et al., 2016). The affinity to ApoE-receptors and lipoprotein subtypes vary functionally between human ApoE isoforms, which differ in two residues: small, phospholipid-rich HDLs are recognized by ApoE2 and ApoE3; while ApoE4 binds to large, triglyceride-rich VLDL. On the other hand, glia, neurons, and brain endothelial cells (BECs) express LDLR and LRP-1 as endocytic receptors, while VLDL receptor (VLDLR) and LRP8/ApoE receptor 2 (ApoER2) function as signaling receptors. In addition, lipidation grade of ApoE can also modify the specificity of its ApoE-receptor binding (El Gaamouch et al., 2016). Thus, in addition to regulating cholesterol/lipid homeostasis in the CNS, ApoE modulates multiple mechanistic pathways that collectively affect neurogenesis, glucose metabolism, synaptic function, cognition, mitochondrial function, neuronal atrophy, neuroinflammation, tau phosphorylation, and metabolism and aggregation of Aβ, which are of relevance for AD.

### Intracellular cholesterol distribution and trafficking between organelles

Once synthesized in the ER, cholesterol is then distributed to different membrane bilayers in the cell. This redistribution in different subcellular compartments occurs by an arrangement between vesicle-mediated inter-organelle cholesterol transport and protein-mediated (i.e., non-vesicular) monomeric cholesterol transfer through the aqueous cytoplasm (Zhang and Liu, 2015). Vesicular trafficking involves the incorporation of cholesterol into the membranes of transport vesicles that migrates along the cytoskeletal tracks when metabolic energy is available. Non-vesicular transport, however, requires sterol transfer proteins, which pack cholesterol in a hydrophobic pocket that shuttles through the aqueous cytosol between membranes, especially those that are anchored together by specialized proteins (Mesmin et al., 2013).

A number of proteins, including members of steroidogenic acute regulatory protein (StAR)-related domain lipid transfer (START) family, and oxysterol-binding protein (OSBP)-related proteins (ORPs), are thought to contribute to the non-vesicular transport of cholesterol within the aqueous environment of the cytosol, and between organelles and the PM (Figure 2; Lev, 2010; Mesmin et al., 2013). Human START proteins are a family of 15 members, sharing a conserved C-terminal with a 210-residue hydrophobic binding pocket able to accommodate a single lipid molecule that ingress and egress by an α-helix that operates as a “lid” (Thorsell et al., 2011). Among them, the members that bind sterols are STARD1, STARD3, and STARD4 subfamily (Soffientini and Graham, 2016; Elustondo et al., 2017). The second group of lipid-binding/sensing proteins includes the ORPs that have a conserved C-terminal OSBP-homology domain, and bind cholesterol, ergosterol, oxysterols, phosphatidylinositol-4-phosphate, and/or phosphatidylserine (Olkkonen and Li, 2013). Sterol-binding, sterol-sensing and trafficking proteins influence the expression and activity of sterol-responsive transcription factors, regulating the expression of genes-encoding proteins that synthesize, metabolize and release cholesterol, and direct the trafficking of cholesterol between the PM, mitochondria, Golgi and endosomal/lysosomal compartments, determining the fate of the cholesterol (El Gaamouch et al., 2016; Soffientini and Graham, 2016).

Before it reaches subcellular membrane compartments, externally endocytosed ApoE-cholesterol complex enters late endosome/lysosome (LE/LY) where the ApoE-containing cholesterol ester is hydrolyzed by acid lipase to generate free cholesterol, which is then distributed primarily to the PM as well as to the ER via Niemann-Pick type C1 (NPC1) and C2 (NPC2) protein-dependent mechanisms (Figure 2; Fernández-Checa, 2015). NPC2 first binds the hydrophobic side chain of unesterified cholesterol and hands over the molecule to the N-terminal domain of NPC1, which inserts cholesterol into the lysosomal membrane. Although, the precise mechanism by which NPC1 mediates cholesterol egress from LE to the ER and PM is not fully understood, recently it has been proposed that NPC1 seems to promote a cholesterol-enriched lysosome-related organelle (LRO) formation able to move along actin filaments in a Rab8- and myosin-5-dependent manner toward the PM (Luo et al., 2017). Furthermore, although oxysterol binding proteins such as OPR5 work directly with NPC1, recent findings uncovered an interaction between RIDα and ORP5 that could bypass NPC1 function in the presence of NPC2, which represents a minor route for cholesterol egress from lysosomes (Cianciola et al., 2013). An excess of PM's cholesterol triggers its retrograde transport to the ER by a STARD4, ORP1, and ORP2-dependent process (Luo et al., 2017). In addition, the transfer of cholesterol from LE/LY-to-ER seems to occur by key components of some ER-LE membrane contact sites (MCS) bridging complexes, which include cholesterol-binding proteins such as ORP1L, ORP5, and STARD3 (Luo et al., 2017). Thus, an excess of cholesterol content in ER triggers a feedback mechanism that regulate cholesterol homeostasis at the transcriptional and post-transcriptional levels, including blockage of SREBP/SCAP ER-to-Golgi transport, proteasomal degradation of HMGCR, and activation of ACAT1 to produce cholesteryl esters stored in lipid droplets (Figure 2). In addition, ORPs act as transporters and/or lipid sensors at MCS, exemplified by the exchange of cholesterol and PI4P at the ER-Golgi contact site (Olkkonen and Li, 2013; Goto et al., 2016).

### Membrane lipid rafts

**Figure 3:**
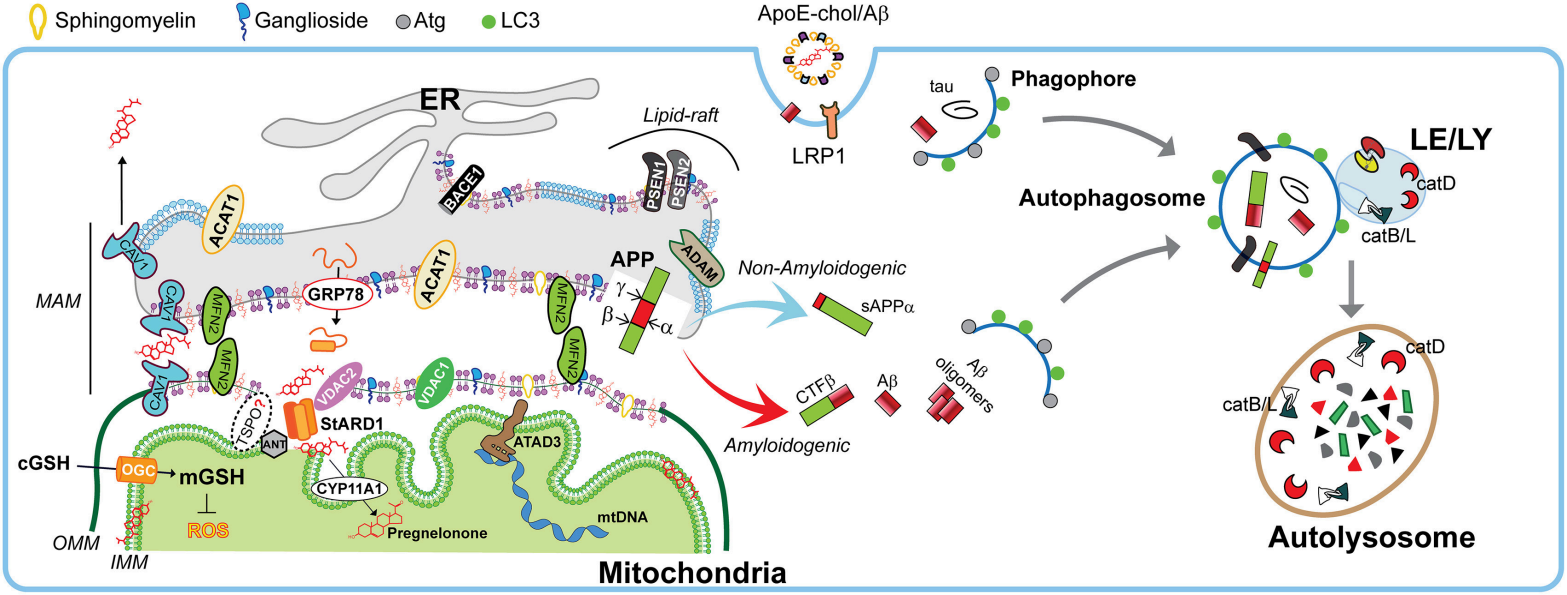
Physiological role of MAMs in mitochondrial function and autophagy. Schematic representation of membrane-associated ER membrane (MAM) as an intracellular lipid-raft subdomain of ER (thicker region) enriched in cholesterol, anionic phospholipids (purple), sphingomyelin, and ganglosides, compared to the bulk ER (thinner region). Relevant components that connects with mitochondria both physically and biochemically are described in the text. Right panel shows the endocytic-autophagic-lysosomal system responsible to degrade and recycle proteins. In physiological conditions, the components that manage the processing of amyloid precursor protein (APP) seems to be spatially segregated in separated membrane domains (raft and non-raft, and even possibly in different neuronal organelles), which limits the amyloidogenic APP processing (red arrow) and maintain the non-amyloidogenic APP processing (light blue arrow). This segregation depends on membrane fluidity, which in turn is mediated by cholesterol levels. Moreover, the GRP78/VDAC2-dependent activation of StARD1 ensure the rapid OMM-to-IMM cholesterol delivery needed to maintain the very strict limit in the IMM-cholesterol levels that are critical to ensure the intra-mitochondrial antioxidant defense (mGSH), a proper pregnenolone production, and a correct mitochondrial DNA (mtDNA) function. Moreover, the specific integral MAM component CAV1 (light blue drawing) seems to not only modulate MAM-cholesterol contents by the cholesterol efflux out of MAM toward PM, but also increase α-secretase activity. On the other hand, healthy neurons execute highly efficient autophagy of either intracellular tau and Aβ peptides or endocytic Aβ. Autophagy induction begins with phagophore formation, which required LC3 and Atg proteins. The recruitment of cytosolic proteins and organelles ends when is formed the autophagosome, which fuse with lysosomes. The resulting autolysosome degrade enclosed cytosolic content by acidic hydrolases such as the neuron-enriched cathepsin D (catD) or cysteine cathepsins B and L (catB/L).

Once distributed in organelles, intracellular cholesterol not only generates a semipermeable barrier between cellular compartments that regulates membrane fluidity, it also induces the formation of cholesterol-enriched microdomains of the membrane called membrane “lipid-rafts.” Besides cholesterol, lipid-rafts are characterized by the presence of sphingolipids and specific scaffolding proteins that act as platforms for signal transduction, cytoskeletal organization, and vesicular trafficking (Egawa et al., 2015). Of particular relevance for lipid-rafts are the presence of caveolins (CAV), essential components of caveolae, which exhibit both scaffolding- and cholesterol-binding functions. Non-caveolar pools of CAVs are presents in myriad locations, including endosomes, ER, lipid droplets, and mitochondria (Bosch et al., 2011). CAVs regulate many receptors, including neurotransmitter receptors (NMDA and AMPA receptors), signaling proteins that trigger the production of cAMP (G protein-coupled receptors, adenylyl cyclases, and phosphodiesterases), and receptor tyrosine kinases involved in growth (e.g., Trk). Moreover, components that control cytoskeletal dynamics (e.g., RhoGTPases and actin binding proteins) interacts with CAVs as well (Egawa et al., 2015). In addition, the presence of CAVs is thought to promote the stability of lipid-rafts (Colin et al., 2016). In particular, CAV1 is a mitochondrial ER-associated membrane (MAM)-resident protein that binds cholesterol with high affinity and is transcriptionally upregulated by cholesterol levels. CAV1 seems to promote cholesterol efflux out of the ER toward PM (Figure 3), reducing the availability of cholesterol in MAM, hence limiting its trafficking to mitochondria (Bosch et al., 2011).

**Figure 4:**
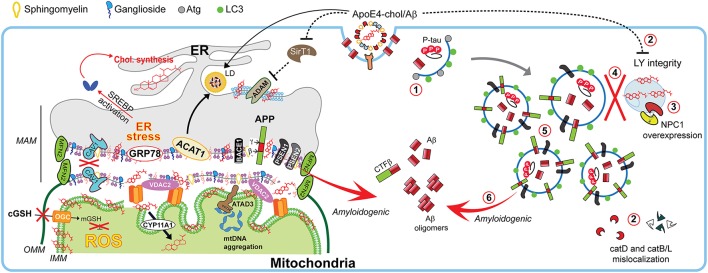
Impact of cholesterol on MAM dynamics, mitochondrial fitness, and autophagic processes involved in Alzheimer disease. In Alzheimer disease (AD), dysregulation of cholesterol homeostasis leads to a reorganization of ER-membrane domains and co-locating the amyloidogenic components at the MAM to increase Aβ biogenesis. Schematic representation of the endocytic-autophagic-lysosomal axis involved in AD, which includes: (1) decreased expression and activity of autophagy-inducing proteins such as Atg; (2) ApoE4-induced disruption of lysosomal integrity and mislocalization of lysosome proteases; (3) increased NPC1 overexpression in disease-affected brain areas; (4) defective lysosomal fusion, resulting in concomitant (5) high levels of autophagic and endosomes vacuoles containing undigested pathological proteins such as PSEN1 and APP capable of (6) generating Aβ peptides.

Age-dependent loss of cholesterol from PM leads to loss of lipid-rafts, impacting in decreased presynaptic vesicle fusion and disruption of neurotransmitter release. Both events are related with different forms of neurodegeneration (Egawa et al., 2015). Hence any disturbances in lipid-raft's cholesterol levels potentially contribute to pathogenesis of neurodegenerative diseases. Indeed, most of the components involved in the amyloidogenic pathway (described below), such as APP, the proteolytic enzymes BACE1 and presenilins (PSEN1/2), Aβ, and γ-secretase activity are enriched in lipid-rafts (Arbor et al., 2016; Colin et al., 2016), particularly in non-PM's lipid-rafts (Schon and Area-Gomez, 2013). Moreover, in physiological conditions, there are neuronal mechanisms that manage the spatial segregation of amyloidogenic machinery in separate membrane domains, and even has been suggested an organelle segregation in the neurons (Das et al., 2013). Cholesterol can regulate APP accumulation in lipid-rafts and amyloidogenesis since C99 fragment of APP (product of APP cleavage by BACE1) exhibits a binding site for cholesterol, which is immediately adjacent to α-secretase cleavage site (K687) and the presence of cholesterol in membrane-buried GXXXG motif of C99/APP can reduce non-amyloidogenic APP processing to diminish the levels of α-secretase-cleaved soluble APP (sAPPα), which is neurotrophic and neuroprotective (Barrett et al., 2012; Yang et al., 2014). Moreover, the α-secretase activity that depends on membrane fluidity, is reversibly modulated by cholesterol (Yang et al., 2014), which inhibit the secretion of sAPPα (Figure 4). This increased amiloydogenic APP processing by increased cholesterol-mediated loss of membrane fluidity and the depletion of mitochondrial GSH (mGSH) by a similar cholesterol-mediated disruption in mitochondrial membrane order accelerate neurodegeneration by increasing Aβ-induced cell death and oxidative stress (Barbero-Camps et al., 2014), as discussed below.

Interestingly, lipid-rafts components can undergo intracellular re-localization via ER-MAM and microtubular network allowing the presence of raft-like microdomains in mitochondrial membranes, which are enriched not only in cholesterol but also in gangliosides (although with lower content than PM), and relatively poor in phospholipids (Vance, 2014). In fact, PSEN1/2 and γ-secretase activity are localized predominantly at the MAM in concordance with the reported localizations of Aβ and PSEN1 in mitochondria (Area-Gomez and Schon, 2016). This evidence supports the idea that an intracellular APP pathological processing could occur at MAM in addition to the PM (Figure 4).

### Mitochondrial cholesterol trafficking

Given the fact that the ER makes extensive MCS with other organelles such as PM, Golgi, and mitochondria, it is likely that cholesterol can move from ER to these downstream destinations. Indeed, the ER-subdomain of MAMs physically interact with outer mitochondrial membrane (OMM), defining a specific suborganelle characterized by unique biochemical properties and specialized functions that involve both organelles (Figure 3). These actions are dependent on specific biophysical properties of MAM, which are determined by the dynamics of protein/lipid composition, and functional alterations in this particular interface are associated with a number of neurodegenerative disorders (see below). Among others, MAM have been shown to participate in multiple pathways, including calcium homeostasis (e.g., IP3 receptors); phospholipid metabolism (e.g., phosphatidylserine synthase 2, PSS2); ER stress (e.g., GRP78), and cholesterol metabolism (e.g., ACAT1 and HMGCR; Vance, 2014; Area-Gomez and Schon, 2016, 2017; van Vliet and Agostinis, 2017). Interestingly, *in vitro* reconstitution assays showed that among ER membranes, MAMs have higher cholesterol content, which is relevant to the association between ER and mitochondria (Area-Gomez et al., 2012). Furthermore, mitochondrial-related proteins such as adenine nucleotide transporter (ANT) at inner mitochondrial membrane (IMM) and voltage-dependent anion channel (VDAC) at OMM have also been found in MAM, supporting the idea that cholesterol could move from ER to mitochondria via a TSPO/VDAC/STARD1/ANT-dependent mechanism (Figure 3). This is based on observations indicating an interaction between ANT, VDAC, and TSPO (Allouche et al., 2012; Vance, 2014; Stocco et al., 2017), and the fact that STARD1 is first loaded onto the MAM to interact with VDAC2 for its processing to mature active form at the OMM (Prasad et al., 2015). However, the role of TSPO in mitochondrial cholesterol trafficking is under debate. Recent evidence using genetic models, such as loxP-Cre-based global TSPO deletion (*Tspo*^−/−^) or Leydig cell-specific TSPO conditional knockout (*Tspo*^cΔ/Δ^; Morohaku et al., 2014), and CRISP/Cas9-based deletion of TSPO in the MA-10 mouse Leydig cell line (MA-10^*Tspo*Δ/Δ^; Tu et al., 2015) have shown a minimal effect of TSPO ablation on steroidogenesis. Most surprisingly, it was determined that human adrenal H295R cell line did not express TSPO, even though these cells are competent in the synthesis of steroids (Tu et al., 2014). Since the expression of TSPO in STAR-deficient mice is unable to compensate the mitochondrial cholesterol transport deficit (Caron et al., 1997; Ishii et al., 2012) and that isolated *Tspo*-deficient mitochondria exhibit a normal mitochondrial cholesterol import (Banati et al., 2014), it is conceivable that TSPO plays a minor role in mitochondrial cholesterol import for steroidogenesis, questioning the TSPO-STARD1 interaction (Selvaraj and Tu, 2016).

Among subcellular membranes, mitochondrial membranes and particularly the IMM, are cholesterol-poor. However, cholesterol must reach at IMM for the synthesis of neurosteroids in the brain, which are necessary for normal stimulation of GABAergic currents, Purkinje cells's response modulation to excitatory amino acids and the enhancement of memory function (Stocco et al., 2017). StAR-dependent delivery of cholesterol from the OMM to the IMM governed by STARD1 is the rate-limiting step for steroidogenesis by facilitating generation of pregnenolone (precursor of all steroids) from cholesterol through metabolism by cytochrome p450scc (CYP11A1, Figure 3; Elustondo et al., 2017; Stocco et al., 2017). In steroidogenic cells (including specialized neurons), the very low IMM-cholesterol levels enable a faster modulation of steroidogenesis by the rate of the OMM-to-IMM transport of cholesterol rather than enzymatic activity-dependent pathways. However, this process implies that even small changes in cholesterol levels can strongly modify the biophysical/functional features of IMM, which sensitizes mitochondria to alterations in cholesterol content (Elustondo et al., 2017).

Besides ER-derived cholesterol import, LE/LY-derived cholesterol also can be imported into mitochondria in a process involving another START domain containing transport protein, STARD3 (also known as MLN64 and component of ER-LE MCS; Figure 2; Charman et al., 2010). STARD3 N-terminal domain is anchored in the LE membrane (Alpy and Tomasetto, 2006) and its cholesterol-binding START C-terminal domain facing the cytosol (Alpy et al., 2001). Interestingly, STARD3 is present in LE containing cholesterol transporter ABCA3, which can recycle cholesterol to the PM and other membranes (van der Kant et al., 2013). Recently, Balboa and coworkers have reported that overexpression of STARD3 in both normal and NPC1-deficient hepatic cells, increases mitochondrial cholesterol content, resulting in mGSH depletion and mitochondrial dysfunction (Balboa et al., 2017).

In addition, emerging evidence points to the existence of a close relation between ER and lipid droplets, and lipid droplet-derived cholesterol could traffic to mitochondria through the ER (Barbosa and Siniossoglou, 2017). Moreover, recently it has been proposed that soluble N-ethylmaleimide sensitive factor attachment receptor (SNARE) proteins can facilitate the interaction of lipid droplets and mitochondria (Lin et al., 2016). Thus, LE/LY and ER seem to act as the source of mitochondrial cholesterol, although the specific contribution of cholesterol originating from each organelle is not known.

### Cholesterol and mitochondrial DNA

Moreover, in addition to the regulation of membrane physical properties, cholesterol content in the IMM can be relevant for mitochondrial DNA (mtDNA) organization. A proper mitochondrial DNA (mtDNA) transcription/replication is ensure by its association with protein forming complexes called nucleoids, which include the mtDNA polymerase polG and the mtDNA helicase Twinkle. Both proteins are part of a membrane-associated platform. Recent findings have indicated that this platform is associated with cholesterol-enriched membrane structure in close association with ER (Figure 3; Gerhold et al., 2015). Therefore, as cholesterol co-sediments with mtDNA, hence it is conceivable that perturbed cholesterol homeostasis could disrupt mtDNA replication. Indeed, recent findings using human fibroblasts from patients with deletions at ATPase family, AAA domain-containing protein 3 (ATAD3, a raft-like domain resident of MAM and an enhancer of hormonal-induced steroidogenesis) described localized mtDNA aggregation, which translated in impaired distribution and slowed down mtDNA synthesis (Desai et al., 2017). Interestingly, treatment of normal fibroblasts with inhibitors of cholesterol synthesis, trafficking (pravastatin, U18666A) or with soluble cholesterol induced similar features resembling the ATAD3-lacking phenotype. Moreover, similar observations were found in fibroblasts from patients with NPC disease (discussed below; Desai et al., 2017), suggesting that a strict limit in the IMM-cholesterol levels is necessary to ensure correct mtDNA function. Thus, disruption in mitochondrial cholesterol trafficking and distribution can have far-more reaching consequences other than altering intramitochondrial antioxidant defense mechanism and physical properties, contributing to mitochondrial dysfunction through impaired generation of mtDNA-encoded subunits of the respiratory chain complexes, which impact in cell physiology, energetics and survival, culminating in neurodegeneration.

## Role of intracellular cholesterol in neurodegeneration

### The ups and downs of cholesterol in neurodegeneration: the case of Alzheimer disease

Given the key role of cholesterol in the regulation of membrane biophysical properties and cell functions through modulation of signaling pathways, alterations in cholesterol homeostasis have been associated with the disruption of brain functions and onset of major neurodegenerative diseases (for more details see Tables 1–**3**). However, while there has been cumulative data linking increased total cholesterol levels with neurodegeneration, there is also evidence indicating the association for decreased total cholesterol levels with neurodegenerative disorders. This conundrum is well-illustrated in the case AD and although alterations in cholesterol homeostasis have been linked to neurodegenerative disease, however, in AD whether total cholesterol levels are upregulated or downregulated is controversial (Table 1). As briefly mentioned above, amyloidogenic APP processing into toxic Aβ peptides occurs in the cholesterol-enriched lipid-rafts domains of the PM. One of the first lines of evidence that brain's Aβ production can be influenced by cholesterol came from observations pointed that cholesterol-enriched diet is able to increase the amyloidosis in rabbit hippocampal neurons (Sparks et al., 1994). Subsequent data from transgenic mice model designed to deposit cerebral Aβ showed that diet-induced hypercholesterolemia leads to increment in both Aβ deposition and amyloid plaque formation (Refolo et al., 2000), in line with findings in mice fed diets enriched in cholesterol (Shie et al., 2002; Thirumangalakudi et al., 2008). Although these observations linking dietary cholesterol intake to AD are puzzling in view of the negligible rate of cholesterol transport from the periphery into the brain due to intact BBB, it has been shown that BBB permeability is impaired in AD (Ujiie et al., 2003). Consistent with the role of cholesterol in AD pathogenesis, hypercholesterolemia not only correlates with Aβ deposition, but also is a risk factor for AD, while statins intake confers a lower incidence of AD (Notkola et al., 1998; Wolozin et al., 2000; Martins et al., 2009). Moreover, several genes that encodes to protein-related cholesterol homeostasis, including ApoE, CYP46A1, ACAT1/SOAT1, the cholesterol efflux transporters ABCA1 and ABCA7, and LRP1 have been linked to the risk, development or progression of AD (Chen et al., 2008; Kim et al., 2009; Di Paolo and Kim, 2011). Intriguingly, despite these findings for the association of increased cholesterol to AD, there is evidence that decreased cholesterol levels are associated with and promote AD. For instance, previous studies showed a correlation between AD-brain cholesterol content and the reduction of fluidity on the hydrocarbon core region of hippocampal membranes of AD-patients; moreover, respect to controls, the temporal gyrus but not the cerebellum of AD-patients showed a reduction of both cholesterol/phospholipids mole ratio and total cholesterol levels (Mason et al., 1992; Eckert et al., 2000). In addition, in comparison with control subjects decreasing of 24-OHC levels in the frontal and occipital cortex of AD-patients have been reported with an increment of mevalonate pathway's inhibition rather than decreased production of Aβ and amyloid plaques (Heverin et al., 2004; Cole et al., 2005). Furthermore, in the context of examining the homeostasis of lysosomal cholesterol through NPC1, Kagedal and co-workers described decreased cholesterol levels in the hippocampus but not cerebellum of patients with AD compared to control subjects (Kagedal et al., 2010). Thus, these findings illustrate the lack of correlation between total cholesterol levels in brain and AD.

**Table 1:** Selected findings of cholesterol metabolism alterations in Alzheimer disease models.

**Alterations in cholesterol homeostasis**	**Event associated**	**Experimental model**	**References**
Increased plasma cholesterol levels	Increased amyloidogenesis compared to control	Rabbits cholesterol-rich diet	Sparks et al., 1994
Membrane cholesterol depletion	Increased sAPPα secretion Decreased Aβ peptides secretion Increased membrane fluidity by methyl-β-cyclodextrin Impaired internalization of APP by methyl-β-cyclodextrin Increased ADAM10 expression by lovastatin	SH-SY5Y cells treated with lovastatin or methyl-β-cyclodextrin	Kojro et al., 2001; reviewed in Yang et al., 2014
Increased brain ApoE expression	Positive correlation between plasma cholesterol levels and brain Aβ levels	APP/PS1 Tg mice fed HC-diet Primary mouse glial cells	Petanceska et al., 2003
Elevated hippocampal StAR protein levels (neurons and astrocytes)	Probable role of StAR in AD-pathogenesis since its localize to NFTs, neuropil threads and dystrophic neurites	Post-mortem hippocampal and cortical tissues from 16 AD patients	Webber et al., 2006
CE accumulation	Increased ACAT1 expression Decreased BACE1 expression Increased Aβ40 and Aβ42 peptides	Skin fibroblast from AD patients	Pani et al., 2009
Increased plasma cholesterol levels	Impaired spatial learning and memory Decrease in the mitochondrial complexes I and II activities in the cortex Decrease in mitochondrial glutathione levels Increase in the thiobarbituric acid-reactive substances levels Imbalance between glutathione peroxidase/glutathione reductase activities in the cerebral cortex	*LDLr* ^(−/−)^ mice fed cholesterol-rich diet	de Oliveira et al., 2011
Changes in intracellular cholesterol and CE content Modified expression of LXR-targets genes (ACAT1/2, ABCA1, HMGCR, SREBP2) Reduced cholesterol biosynthesis Increased ApoA-I-dependent cholesterol efflux	Modulation of cholesterol metabolism by LXR-ligands or cholesterol modulates APP processing Simvastatin-dependent enhanced of non-amyloidogenic sAPPα secretion	pBCECs treated with cholesterol or oxysterols or simvastatin	Schweinzer et al., 2011
Changes in expression of ApoE isoforms	BBB breakdown by lack apoe and increased by APOE4 APOE4-dependent BBB disruption by activating CypA-NFκB-MM9 pathway in pericytes Astrocytes-secreted APOE3 protect BBB by suppression CypA-NFκB-MM9 pathway via LDLr	*Apoe* ^(−/−)^ mice APOE2- or 3- or 4-TR mice *apoe* ^(−/−)^GFAP-APOE2 or 3 or 4 mice	Bell et al., 2012
Mitochondrial cholesterol loading	Aβ-induced ER stress Aβ-induced mitochondrial GSH depletion Accelerated APP processing and Aβ accumulation in hippocampus Increased neurodegeneration resulting in accelerated cognitive deficits	APP/PS1 Tg mice overexpressing SREBP2	Barbero-Camps et al., 2013
Increased mitochondrial cholesterol content in hippocampus Increased cholesterol synthesis	Age-dependent increasing of active SREBP2 and HMGCR expression Increased ABCA1 expression Decrease levels of INSIG-1 Depletion of GSH in brain mitochondria	APP/PS1 Tg Aβ_42_-treated SH-SY5Y cells	Barbero-Camps et al., 2014
Hypolipidation of ApoE4 ApoE4 levels lower than ApoE3 levels in plasma Decreased ABCA1 activation	ApoE4-driven hippocampal accumulation of Aβ42 and hyperphophorylated tau ApoE4-dependent early cognitive impairments ApoE4-induced synaptic impairments	ApoE3/4-TR mice	Boehm-Cagan and Michaelson, 2014
Increased ApoE4 lipidation and clearance Increased hippocampal expression of ABCA1 and ABCG1	Reversal of the ApoE4-driven neurodegeneration and cognitive deficits	ApoE3/4-TR mice treated with bexarotene or CS-6253	Boehm-Cagan et al., 2016a,b
Increased synthesis of phospholipids and CE Increased lipid droplets	Increased ER-mitochondria communication Increased MAM's function	Mouse hippocampal neurons treated with ApoE4-containing ACM Human fibroblasts treated with ApoE4-containing ACM	Tambini et al., 2016
Lysosomal cholesterol accumulation	Decreased lysosomal degradation capacity Increased lysosomal accumulation of NPC1/2 Downregulated expression of ABCA1 Accumulation of BACE1 and APP-CTFs	PADK-treated SH-SY5Y cells MEFs from CatB^(−/−)^L^(−/−)^ mice	Cermak et al., 2016
Lysosomal cholesterol accumulation	Hippocampal increase of β-hexosaminidase A Increased cathepsin D expression in cortex and hippocampus Cytosolic localization of β-hexosaminidase A and cathepsin D in the hippocampus and cortex	T2D db/db mice	Sims-Robinson et al., 2016
Lysosomal cholesterol accumulation	Increased of APP holoprotein and its cleaved products α-, β-, η-CTFs Increased activities of ADAM10, BACE1, and γ-secretase complex Decreased clearance rate of APP-cleaved products Partial accumulation of APP-cleaved products in LE/LY	U18666A-treated rat primary cultured astrocytes	Yang et al., 2017b
Increased brain levels of NPC1, ABCA1, and cathepsin D and B	Short-term CYCLO-dependent amelioration of lysosonal Aβ accumulation Short-term CYCLO-dependent induction of autophagy Increased lysosomal proteolytic activity by short-term CYCLO treatment Short-term CYCLO-dependent increasing of cathepsin D activity	CYCLO-treated TgCRND8 mice	Yang et al., 2017a
Changes in membrane lipid composition	Disrupted hippocampal autophagosome-lysosome fusion Short-term CYCLO-dependent autophagosome accumulation of LC3, SQSTM1/p62 , and ubiquitinated substrates	CYCLO-treated TgCRND8 mice	Yang et al., 2017a
Lipid droplets accumulation near to ER and mitochondria	APP, APP-CTFs, and Aβ localizated at ER, mitochondria, and MAMs Active forms of BACE1, α- and γ-secretase presents at MAMs Increased ER-mitochondria contact sites APP overexpression and processing, and Aβ production is likely linked to lipid droplet formation	SH-SY5Y cells expressing mutAPP APP/PS1 AD mice	Del Prete et al., 2017
Inhibition of cellular cholesterol synthesis Decreased desmosterol, lathosterol, and lanosterol levels	Increased both expression and basolateral secretion of apoJ Increased expression of APP and ADAM10 Reduced expression of BACE1 Increased levels of CTFs and sAPPα Increased secretion and reduced uptake of Aβ oligomers Reduced APP protein and Aβ oligomers levels by siRNA-mediated apoJ kockdowning Increased expression of apoJ and LRP1 by Aβ(1-40)-treatment	Simvastatin-treated pBCECs	Zandl-Lang et al., 2017
Increased levels of LRP1/2 Decreased apoJ protein levels	Reduction levels of CTFs by decreased APP processing	mBCECs from simvastatin-treated 3x Tg AD and Non-Tg mice	Zandl-Lang et al., 2017

*ACM, astrocyte-conditioned medium; ADAM10, A disintegrin and metalloproteinase domain-containing protein 10; APP, amyloid precursor protein; sAPPα, secreted APP-α; BACE, b-site APP cleaving enzyme; BCECs, brain capillary endothelial cells; CE, cholesteryl ester; CTF, C-terminal fragment; CYCLO, 2-hydroxypropyl-β-cyclodextrin; ER, endoplasmic reticulum; GSH, glutathione; INSIG-1, insulin-induced gene-1; LE/LY, late endosomes/lysosomes; LXR, liver X receptor; mBCECs, murine BCECs; MEFs, mouse embryonic fibroblasts; pBCEC, porcine BCECs; ROS, reactive oxygen species; Tg, transgenic; TR, target replacement*.

Besides AD, homeostasis disturbance in the cholesterol biosynthesis has been also associated with rare hereditary diseases. Indeed, early life impairment brain function is associated to mutations of genes related with cholesterol synthesis. For instance, mutations in *DHCR7* within the Kandutsh-Russell pathway (Figure 1) result in Smith-Lemli-Opitz syndrome (SLOS), a disease characterized by low cholesterol levels in the brain that primarily cause intellectual disabilities, psychiatric problems and delayed maturation of both motor and language skills (Segatto et al., 2014). On the other hand, dysfunction of last step of the astrocytes-predominant Bloch pathway (i.e., enzyme-mediated reduction of desmosterol to cholesterol) by mutations in *DHCR24* gene results in desmosterolosis (Figure 1), also characterized by low brain's cholesterol content related with a generalized malformation of brain's structures, such as ventricular enlargement, thinning of white matter and seizures, defective corpus callosum, microcephalia, and hydrocephalia (Schaaf et al., 2011). Whether this disease is causally linked to reduced cholesterol levels or increased desmosterol remains to be established. In addition, other neurological diseases associated with disturbance of cholesterol biosynthesis include the Rett syndrome (RS), a neurological disorder caused by mutations in the X-linked *MECP2* gene with clinical features that include defects in motor control, cognitive skills, and social interactions such as intellectual incapacities, and autistic behavior. RS has been related with changes in cholesterol metabolism since HMGR, squalene epoxidase, and CYP46A1 are altered in Mecp-2 knock-out mice in an age-dependent manner (Martin et al., 2014). In the following sections we will briefly summarize the role of intracellular cholesterol pools, in neurodegeneration.

### Lysosomal cholesterol in Niemann Pick type C disease, Alzheimer disease, and Parkinson disease

Consistent with its key role in intracellular cholesterol trafficking, alterations in the molecular machinery involved in the release of cholesterol from LE/LY have an impact in brain pathophysiology. For instance, Rab8 GTPase, which promotes the LE/LY to PM cholesterol efflux via LRO (Figure 2) and participates in polarized neurite growth and in the dynamics of glutamate receptors in postsynaptic compartment (Ng and Tang, 2008), has been involved in α-synuclein aggregation and reduced toxicity in cellular models for PD (Yin et al., 2014). In addition, alterations in release of the LE/LY cholesterol by mutations in NPC1/2 proteins cause Niemann-Pick type C (NPC) disease (Figure 2), a progressive and fatal visceral, neurological and psychiatric disorder characterized by increased accumulation of unesterified free cholesterol and other lipids (e.g., glycosphingolipids) predominantly in LE/LY in NPC patient fibroblasts and in axons of cultured neurons with reduced cholesterol levels in the PM. Besides brain pathology, some patients with NPC disease exhibit liver disease and hepatosplenomegaly and like neurons, hepatocytes from NPC null mice exhibit increased cholesterol accumulation and sphingolipids (Mari et al., 2006; Torres et al., 2017). Purkinje cells in the cerebellum are the most vulnerable neurons in NPC disease, accounting for the characteristic ataxia of NPC patients (Malnar et al., 2014). Moreover, not only synaptic vesicle composition and morphology are affected, but also endosomal organelle transport, leading to neuronal loss in NPC disease (Schultz et al., 2016). Animal models for NPC disease (i.e., NPC knockout mice) mimic most of the pathological features of NPC patients and display loss of neurons rather than astrocytes as the key determinant of the neurodegeneration in NPC disease (Schultz et al., 2016). Moreover, increased levels of cholesterol in mitochondria from brain and hepatocytes of *Npc1*^−/−^ mice have been reported as well (Yu et al., 2005; Fernandez et al., 2009), highlighting the close relationship between both organelles in cholesterol trafficking. Although there are considerable neuropathological differences at the anatomical level between NPC and AD, both diseases share many parallels, including progressive neurodegeneration, LE/LY abnormalities, cholesterol accumulation, hyperphosphorylation of tau, APP processing and Aβ accumulation (Figure 4). While there is no evidence of amyloid plaques in human NPC brains, likely due to premature death, several studies with NPC models in cells and mice showed that the amyloidogenic APP processing can be modulated by the disruption of normal cholesterol trafficking observed in NPC (Malnar et al., 2014). Indeed, compared to CHO-*wt* cells, CHO-*NPC*^−^/^−^ cells exhibit increased levels of BACE1-generated APP metabolites (sAPPβ and C99) and an intracellular Aβ accumulation, which was reversed by cholesterol depletion, indicating that the enhanced BACE1 cleavage of APP occurs as a consequence of cholesterol accumulation rather than NPC1 loss (Malnar et al., 2010). Although expression of NPC1 has been poorly characterized in AD, Kagedal and coworkers described in the hippocampus and frontal cortex of AD an increased expression of NPC1 both at the mRNA and protein levels compared to control subjects, with no changes in cerebellum (Kagedal et al., 2010). Similarly, 12-month-old APP/PS1 transgenic mice showed increased hippocampal murine NPC1 transcripts compared to age-matched wild type mice (Kagedal et al., 2010). Thus, these findings imply the impossibility of accumulation of lysosomal cholesterol in AD, and suggest that the lysosomal deficiency that contributes to impair Aβ degradation in AD possibly occur by mechanisms independent of lysosomal cholesterol accumulation. Among the parallels existing between NPC disease and AD, the mitochondrial cholesterol loading is the most common link between these diseases rather than lysosomal cholesterol accumulation (Webber et al., 2006; Fernandez et al., 2009; Torres et al., 2017).

To assess the impact of lysosomal cholesterol accumulation in APP processing, Aβ generation and AD pathology, Maulik et al. generated a mouse line (ANPC), which expressed mutant human APP in the context of genetic deletion of NPC1 (Maulik et al., 2015). The phenotype included increased cholesterol accumulation, leading to enhanced APP C-terminal CTF fragments and Aβ generation, as well as decreased clearance of peptides and increased γ-secretase activity. Cholesterol sequestration in ANPC mice impaired endocytic-autophagic-lysosomal but not proteasome clearance of APP-CTFs/Aβ peptides, and vulnerable brain regions of ANPC mice exhibited increased oxidative stress and susceptibility to H_2_O_2_-induced toxicity. In line with these effects of increased cholesterol in lysosomes, extraction with 2-hydroxypropyl-cyclodextrin (CDX) in AD models revealed a dual effect of CDX in modulating the mechanisms involved in AD pathology (Yang et al., 2017a). While CDX enhanced lysosomal activity, it impaired autophagosome-lysosome fusion resulting in defective autophagy and clearance of Aβ fragments (Yang et al., 2017b). Together, these findings strongly suggest that unlike NPC disease, the accumulation of cholesterol in lysosomes does not seem to be a characteristic feature of AD and that prolonged treatment with CDX should be carefully evaluated to avoid unwanted effects on autophagy. Whether the increase in NPC1 in AD is a compensatory mechanism to stimulate lysosomal cholesterol efflux remains to be determined.

On the other hand, metabolic disorders such as type 2 diabetes mellitus (T2DM), which is a risk factor for AD, are associated with dementia involving several pathophysiological mechanisms (Li and Huang, 2016). For instance, oxidized cholesterol by intrinsic T2DM hypercholesterolemia in db/db mice not only leads to destabilization of lysosomes, but also alterations in the expression and activity in the hippocampus of the lysosomal enzymes, such as cathepsin D (CatD; Figure 4; Sims-Robinson et al., 2016) by an ApoE4-dependent mechanism related to disruption of lysosomal integrity, including the mislocalization of CatD, which contributes to degradation of the antioxidant protein thioredoxin-1 (Persson et al., 2017). However, some authors pointed to cysteine cathepsins B and L (CatB/L; Figure 4) as the main lysosomal enzyme(s) involved in the abnormalities of intracellular cholesterol trafficking related to AD-like features, since unlike CatD, the CatB/L inhibition in SH-SY5Y human neuroblastoma cells by PADK-treatment or its genetic ablation in CatB^(−/−)^/L^(−/−)^ MEFs resembled NPC disease features (e.g., lysosomal dysfunction by accumulation of both cholesterol and NPC1; and downregulation of ABCA1 expression) and promotes amyloidogenesis assessed by BACE1 and APP-CTFs accumulation (Cermak et al., 2016). Nonetheless, it should be noted that CatD mediates the proteolysis of ApoE and both colocalized in AD frontal cortex on neuritic plaques, which in despite above, suggests a plausible role of CatD in the AD pathogenesis through ApoE fragments (Zhou et al., 2006). In fact, recently it has been described that ApoE4 is able to translocate to the nucleus and even acting as a transcription factor given its capability to bind to double-stranded DNA with high affinity (infra nanomolar; Theendakara et al., 2016). For instance, Rao and colleagues reported that ApoE4 specifically represses the expression of Sirtuin 1 (SirT1), a transcriptional activator of the α-secretase ADAM10 that promotes the production of the neuroprotective sAPPα peptide, hence contributing to stimulated Aβ production (Figure 4; Theendakara et al., 2016).

PD is the second most common neurodegenerative disorder after AD and it is characterized by the accumulation of intraneuronal cytoplasmic inclusions of Lewy bodies, caused by the aggregation of α-synuclein. Similar to AD, there is a large body of data linking alterations in total cholesterol levels with neurodegeneration in PD (Table 2). In cellular models of PD, the neurotoxin 1-methyl-4-methylpyridinium (MPP^+^), a mitochondrial toxin, triggered lysosomal cholesterol accumulation preceding cell death (Eriksson et al., 2017). This event was ameliorated by preincubation with U18666A, suggesting that neurons respond to early apoptotic stress increasing accumulation of lysosomal cholesterol to preserve the integrity of lysosomal membrane. Moreover, high cholesterol loading also promotes α-synuclein accumulation, and the treatment with lovastatin reduced MPP^+^-induced cell death by decreasing ROS but did not prevent lysosomal cholesterol increased nor affected α-synuclein accumulation. Thus, these findings indicate a dual role of cholesterol accumulation in PD, protecting against lysosomal membrane permeabilization and cell death, while stimulating the accumulation of α-synuclein. Whether cholesterol accumulates in lysosomes or if the expression of proteins regulating lysosomal cholesterol trafficking is altered in patients with PD remains to be investigated.

**Table 2:** Significant observations relating cholesterol metabolism alterations and Parkinson disease.

**Alterations in cholesterol homeostasis**	**Event associated**	**Experimental model**	**References**
Brain's HMGCoA reductase activity reduced by >50% Brain's cholesterol esterifying activity reduced by 43%	Increased lipid peroxidation Defective mitochondrial respiratory chain complex I activity	Fibroblasts from PD patients	Musanti et al., 1993
Disturbances in lipid droplet formation	Binding of α-syn to lipid droplet surfaces as protection to lipase-mediated lipid hydrolysis PD mutant α-syn that binds to lipid droplets surface do not protects against hydrolysis	HeLa cells overexpressing wt or mutant α-syn Primary hippocampal neurons of WT- or A53T-Tg mice HEK293 cells stably expressing wt or mutant α-syn	Cole et al., 2002
Plasma's HMGCoA reductase activity increase Increase serum digoxin and dolichol Decreased cholesterol:phospholipid ratio in erythrocyte membranes	Reduction of Na^+^-K^+^ ATPase activity in erythrocytes Decreased available ATP by mitochondrial malfunction Plausible decreased synthesis of glutathione by low ATP Probable increasing of glutamatergic excitatory transmission by digoxin Probable increase N-glycosylation of proteins by dolichol Plausible increasing phospholipid degradation and defective membrane formation	Plasma erythrocytes of PD patients	Kurup and Kurup, 2003
Increase of cholesterol and CE levels	Increase of fatty acid esterification by α-synuclein deficiency in astrocytes Decreased uptake of fatty acids 16:0 and 20:4 by α-syn deficiency in astrocytes	Primary cortical astrocytes from WT- or *Scna*^−/−^ mice	Castagnet et al., 2005
Endolysosomal free cholesterol accumulation	Disrupted interaction between GCase with Sap C and lysobisphosphatidic acid-containing membranes	Homozygous N370S mutated human GD fibroblasts	Salvioli et al., 2005; reviewed in Galvagnion, 2017
Increase of oxidative cholesterol metabolites	α-synuclein-induced ROS production	LBD patients patients SH-SY5Y human neuroblastoma cells overexpressing α-syn	Bosco et al., 2006
Whole brain increment of non-myelin associated CE levels	Decreased acyl-CoA synthetase activity by α-syn deficiency Unaltered expression of HMG-CoA reductase, ABCA1, LXR or ApoE	*Scna*^−/−^ mice	Barcelo-Coblijn et al., 2007; reviewed in Paul et al., 2015
Increased ABCA1 expression	LXR-dependent upregulation of α-syn mRNA by treatment with 27-OHC	SK-N-SH neuroblastoma cells MO3.13 olygodendrocyte cells	Cheng et al., 2008
Increased levels of serum total cholesterol	High total cholesterol at baseline is associated with an increased risk of PD	18-years follow-up of cohort included >50000 people without PD	Hu et al., 2008; reviewed in Paul et al., 2015
Higher levels of serum total cholesterol	High total serum cholesterol is associated with a modest slower clinical progression of PD	2-years follow-up of 774 PD patients treated with DATATOP	Huang et al., 2011; reviewed in Paul et al., 2015
Higher levels of serum total cholesterol	No probable association between serum cholesterol and risk of PD	Meta-analysis of 8 studies involving >246000 subjects	Gudala et al., 2013; reviewed in Paul et al., 2015
Higher dietary intake of cholesterol	Higher intakes of cholesterol may reduce risk of PD in men	>14-years follow-up of cohort included >63000 subjects	Tan et al., 2016
Decreased lanosterol levels	Drug-induced ER-to-mitochondria redistribution of LSS Exogenous added lanosterol protects dopaminergic neurons from toxin-induced cell death Exogenous added lanosterol uncouples mitochondria in dopaminergic neurons Exogenous added lanosterol increases axonal mitophagy in dopaminergic neurons	MPTP-injected mice MPP^+^-treated mouse primary dopaminergic cells cultures	Lim et al., 2012; and reviewed in Saeedi Saravi et al., 2017
Accumulation of ACAT1-dependent esterified 24S-OHC	Early ACAT1-dependent lipid droplet formation Induces necroptosis in SH-SY5Y cells and apoptosis of Jurkat cells	SH-SY5Y and Jurkat cells treated with 24S-OHC	Yamanaka et al., 2014; reviewed in Paul et al., 2015
Increase of cholesterol and CE levels	Decreasing GCase activity leads ER stress and accumulation of cholesterol Dysregulation of macroautophagy and CMA and increases α-synuclein protein levels Impaired lysosomal recycling and increased α-synuclein accumulation	N370S mutated human GD fibroblast *Gba1* KO MEFs GBA knockdown in SH-SY5Y cells CBE-treated primary cortical neurons of mouse	Magalhaes et al., 2016; reviewed in Galvagnion, 2017
Increased levels of NCEH-1 in DA cells	Significant protection to α-syn neurotoxicity	*C. elegans* with DA-specific overexpressing α-syn and NCEH-1	Zhang et al., 2017
Lysosomal cholesterol accumulation	Release cathepsin D from the LMP LMP reduced by pre-treatment with U18666A Increased intracellular levels of oligomeric α-synuclein close to lysosomes Increased ROS production and loss of mitochondrial membrane potential	BE(2)-M17 neuroblastoma cells treated with MPP^+^	Eriksson et al., 2017
Mevalonate accumulation	Mitochondrial dysfunction increasing ROS and trigger activation of caspase-3 and -9 Pyroptosis by activation of caspase-1 and neuro-inflammation Impaired production of TGF-β and IL-10 and activation of IL-1β	MKD model: lovastatin-treated SH-SY5Y neuroblastoma cells	Reviewed in Saeedi Saravi et al., 2017
Excess of oxysterols levels	24S-OHC induce apoptosis by: Increase of Ca^+2^ release and ROS, DNA-fragmentation, caspase-3 activation, and Decrease of mitochondrial membrane potential 27-OHC caused: Induction of apoptosis, Reduction of TH levels through inhibition of estrogen receptor β, Overexpression of α-syn via LXR-mediated transcription activation of SCNA 24S-OHC and 27-OHC synergistically induces: Apoptosis, Reduction of TH expression, Increase of α-syn levels	SH-SY5Y cells treated with 24S-OHC and/or 27-OHC	Reviewed in Paul et al., 2015; y Galvagnion, 2017
Increase total cholesterol levels in serum (2.1x) and striatum (2.5x)	DA loss in SN, reduction striatal dopamine levels, and motor impairment Exacerbation of MPTP-induced neurodegeneration and motor impairment Nigrostriatal impaired activity of mitochondrial complexes I and III Increased nigrostriatal generation of hydroxyl radicals Increased MPTP-induced GSH depletion in striatum Enhanced activity of antioxidant enzymes (SOD and catalase)	Normal and MPTP-injected mice fed high cholesterol (5%) diet during 3.5 months	Paul et al., 2017

*6-OHDA, 6-hydroxydopamine; 24S-OHC, 24-hydroxycholesteol; 27-OHC, 27-hydroxycholesterol; α-syn, α-synuclein; CBE, GCase inhibitor conduritol-β-epoxide; CE: cholesteryl ester; CMA, chaperone mediated autophagy; DA, dopaminergic neurons; DATATOP, Deprenyl and Tocopherol Antioxidative Therapy of Parkinsonism; MEFs, mouse embryonic fibroblasts; MPTP, 1-methyl-4-phenyl-1,2,3,6-tetrahydropyridine; GBA, glucocerebrosidase; GCase, glucosylceramidase; GD, Gaucher disease; GSH, reduced glutathione; LBD, Lewy body dementia; LMP, lysosome membrane permeabilization; LSS, lanosterol synthase; LXR, liver X receptor; MPP^+^, 1-methyl-4-phenylpyridinium; MPTP, 1-methyl-4-phenyl-1,2,3,6-tetrahydropyridine hydrochloride; MKD, mevalonate kinase deficiency; NCEH-1, neutral cholesterol ester hydrolase 1; PD, Parkinson's disease; ROS, reactive oxygen species; Sap C, saposin C; Scna, α-synuclein gene; SN, substantia nigra; SOD, superoxide dismutase; Tg, transgenic; TH, tyrosine hydroxylase; WT, wild-type*.

### Mitochondrial cholesterol in Alzheimer disease, NPC disease, and Huntington disease

Mitochondria are cholesterol-poor organelles compared to PM. However, the restricted pool of mitochondrial cholesterol, particularly at IMM, is crucial for the synthesis of neurosteroids in the brain and for physiological GABAergic responses that modulate memory function (Stocco et al., 2017). As mentioned above, the rate of cholesterol translocation from OMM to IMM controls steroidogenesis, indicating that changes in the levels of cholesterol in IMM has a significant impact in the extramitochondrial functions in specialized tissues mediated by the generation of pregnenolone and subsequent steroids derived from the metabolism of cholesterol in the IMM. Moreover, pathological conditions leading to unphysiological cholesterol accumulation in mitochondrial membranes can have a profound effect in mitochondrial function, including defective mitochondrial antioxidant defense. Indeed, we have recently provided evidence for a critical role of mitochondrial cholesterol loading in the aggravation and acceleration of AD through the generation of a novel genetic mouse model of AD characterized by the overexpression of SREBP-2 in the background of APP/PS1 transgenic (APP/PS1/SREBP2; Barbero-Camps et al., 2013). The pathological events in APP/PS1/SREBP2 mice precipitating AD-like symptoms were dependent on mGSH depletion (Figure 4), since *in vivo* replenishment of mGSH with cell-permeable GSH monoethyl ester (GSHee) attenuated neuropathological features of AD in APP/PS1/SREBP-2 mice, including decreased neuroinflammation, cell death and tau-phosphorylation, which led to improvement of cognitive defects (Barbero-Camps et al., 2013). Quite intriguingly, although NPC disease is primarily characterized by increased lysosomal cholesterol, NPC fibroblasts, and *Npc1*^−/−^ mice also exhibit increased mitochondrial cholesterol accumulation and subsequent mGSH depletion (Torres et al., 2017). Similar to the APP/PS1/SREBP2 AD model, NPC1^−/−^ mice display increased expression of StARD1 by an ER stress-independent mechanism, which remains to be uncovered (Torres et al., 2017). Treatment with GSHee restored mGSH levels in brain of NPC1^−/−^ mice and fibroblasts from NPC patients without alterations in the profile of most sphingolipids, resulting in increased life span, improved mitochondrial function and enhanced Purkinje cell survival, underlying the reversal of locomotor deficits. Interestingly, these beneficial effects of GSHee contrast with the failure of N-acetylcysteine (NAC) to improve NPC pathology due to its inability to restore mGSH levels despite significant increase in cytosol GSH pool (Torres et al., 2017). The levels of mitochondrial reactive oxygen species (mROS) are modulated by mGSH and increased mROS levels contribute to mDNA mutations, mitochondrial dysfunction, and cellular death (Ribas et al., 2014). The mitochondrial transport of GSH from cytosol where it is synthesized *de novo* from its constituent aminoacids is dependent on mitochondrial membrane composition and fluidity (Ribas et al., 2014) and accumulation of cholesterol in IMM governed by StARD1 affects biophysical membrane properties, function and antioxidant defense, which overall contribute to the pathology of AD (Figure 4). These findings are in line with the central role of changes in membrane biophysical properties in AD (Yang et al., 2014). Indeed, an increase in membrane fluidity correlates with a rearrangement of cholesterol, sphingomyelin, and proteins-related to APP processing between raft and non-raft domains, shifting APP processing to non-amyloidogenic pathway enhancing sAPPα production (Yang et al., 2014). Moreover, CDX-mediated removal of cholesterol increased membrane fluidity and leads to α-secretase overexpression in neuroblastoma SH-SY5Y cells (Kojro et al., 2001), whereas, higher levels of mitochondrial APP levels in AD-affected brain areas have been found to correlate with mitochondrial dysfunction (Lin and Beal, 2006).

The role of StARD1 in human AD is poorly understood. Increased StARD1 expression in pyramidal hippocampal neurons of AD-patients has been described (Webber et al., 2006). Although the functional consequences of this event in the regulation of mitochondrial cholesterol levels and mitochondrial function were not examined in human AD, enhanced mitochondrial cholesterol loading in AD mouse models has been shown to sensitize neurons to Aβ-induced inflammation and toxicity by depleting mGSH through a mechanism dependent of ER stress, which in turn modulates lipid metabolism (Figure 4; Barbero-Camps et al., 2014). In addition, Aβ-induced ER stress is indirectly involved as an effector of the Aβ-neurotoxicity in early stages of AD (Hoozemans et al., 2012). In fact, treatment with ER stress inhibitors such as TUDCA blocked Aβ-induced mGSH depletion (Barbero-Camps et al., 2014) and the immunoreactivity of the ER stress marker GRP78 correlated with Braak AD-stages in postmortem AD-brains, which also exhibit alterations in proteins levels of the ER stress components (Placido et al., 2014). In line with recent findings in mouse hepatocytes showing that ER stress induces the expression of StARD1 (Fernandez et al., 2013), it is conceivable that Aβ-mediated ER stress triggers mitochondrial cholesterol loading through StARD1 upregulation, which in turn sensitizes to Aβ-induced cytotoxicity by mGSH depletion due to mitochondrial cholesterol accumulation. This link between Aβ-ER stress-StARD1-mitochondrial cholesterol loading-mGSH depletion can be abrogated by chaperones that block ER stress. Overall, while cholesterol may modulate APP amiloydogenic processing and Aβ production, the mitochondrial cholesterol pool emerges as a novel factor in modulating the susceptibility to the noxious effects of toxic Aβ fragments and tau-phosphorylation underlying AD. Due to the restriction of efficient cytosol GSH transport to mitochondrial imposed by mitochondrial-mediated decrease in membrane dynamics, strategies that increase cytosol GSH levels may not be sufficient to boost mGSH levels. Novel GSH precursors, such as γ-glutamylcysteine ethyl ester (γ-GCEE) or S-acyl GSH thioester have been shown to increase total GSH in fibroblasts from patients with AD and protect primary neurons against Aβ-induced cell death (Boyd-Kimball et al., 2005; Zampagni et al., 2012). However, whether these GSH precursors were efficient in increasing mGSH deserves further investigation.

In contrast to AD, there has been very limited research on the role of mitochondrial cholesterol in other major neurodegenerative diseases, such as HD (Table 3), an autosomal-dominant neurodegenerative disorder caused by a CAG expansion in the Huntingtin (htt) gene. For instance, CAV1 knockout mice, which is characterized by increased mitochondrial cholesterol trafficking via MAM and subsequent mGSH depletion, exhibited increased brain damage caused by 3-nitropropionic acid (3NP) treatment (Bosch et al., 2011). 3-NP is a mitochondrial toxin used extensively as a model of HD and its toxicity is associated with oxidative stress. Injection of 3-NP in the striatum of CAV1 null mice caused extensive cell degeneration as revealed by Fluoro-Jade stained serial sections compared to wild type mice. In addition, there is evidence that the multifunctional htt protein intimately interacts with a variety of lipid membranes, and is essential for the normal development of several perinuclear membrane organelles, including mitochondria and the ER (Gao et al., 2016). Moreover, brain-isolated mitochondrial membranes of HD models such as aged Hdh Q111/Q111 knock-in mice and BACHD transgenic rats as well as isolated from striatal STHdh Q111/Q111 cells, exhibited increased membrane fluidity (Eckmann et al., 2014). Intriguingly, however, both models of HD knock-in mice treated with olexosime, a cholesterol-like compound, showed decreasing membrane fluidity in brain-isolated mitochondrial membrane fractions and restored HD-specific changes in mitochondrial membranes. Moreover, BACHD rats treated during 12-months with olexosime showed counteracted the mhtt-induced membrane fluidity increment of brain-isolated mitochondrial membranes. Although, these findings link altered mitochondrial membrane properties with HD and suggest that olexosime may be a potential therapeutic opportunity for HD, whether these effects are mediated by modulating mitochondrial cholesterol levels in OMM o IMM remains to be further investigated.

**Table 3:** Selection of latest relevant findings of cholesterol metabolism alterations in Huntington disease models.

**Alterations in cholesterol homeostasis**	**Event associated**	**Experimental model**	**References**
Reduced both cortex and striatal levels of lathosterol and lanosterol	Plausible reduced activity of DHCR7 enzyme	R6/1 HD mice	Kreilaus et al., 2015
Elevated levels of desmosterol	Plausible reduced activity of DHCR24 enzyme	R6/1 HD mice	Kreilaus et al., 2015
Striatal reduction of 24S-OHC and 27-OHC levels	Aging-dependent increased activity of CYP46A1	R6/1 HD mice	Kreilaus et al., 2015
Reduced brain levels of cholesterol, lathosterol, lanosterol, desmosterol and 7-dehydrocholesterol Reduced brain levels of 24S-OHC and 27-OHC	Reduced expression of HMGCS1 and HMGCR Reduced MBP expression PGC1α transcriptionally regulate the expression of MBP and SREBP2 mutHTT decreased expression of PGC1α and its targets	PGC1α KO mice Primary rat oligodendrocytes knocking-down PGC1α with/without mutHTT R6/2 HD mouse and BACHD rat	Xiang et al., 2011; reviewed in Leoni and Caccia, 2015
Early reduction in striatal lathosterol levels Decreased 24S-OHC synthesis rate per day Striatal reduction in cholesterol content	Decreased striatal daily synthesis rate of cholesterol Reduced striatal mRNA levels for *hmgcr* and *fdft1* by reduced translocation of active SREBP2	Heterozygous Hdh Q175 knock-in mouse	Shankaran et al., 2017
Reduction of lathosterol striatal content by mut HTT Plasma 24S-OHC reduction proportional to disease progression	CAG expansion-dependent reduction of lathosterol only in Q111 and Q175 animals	Heterozygous Q7 (wt), Q20 (wt), Q80, Q111, Q175 knock-in mice	Shankaran et al., 2017
Reduction of lanosterol and lathosterol striatal content associate a mut HTT	Reduction in sterol precursors is inversely proportional to CAG repeat	Hdh (Q7/Q111), (Q111/Q111) knock-in mice	Reviewed in Leoni and Caccia, 2015
Decreased rate of cholesterol synthesis Reduced total cholesterol content	Reduced mRNA levels of HMGCR, CYP51, 7-dehydrocholesterol 7-reductase, and DHCR24	Inducible mutant HTT cell line R6/2 HTT-fragment Tg mice Post-mortem cortical tissue from HD patients Primary neurons from Hdh(Q140/140) mice Immortalized striatal knock-in cell carrying 109Q inserted in mouse *htt* gene Astrocytes from R6/2 HD and YAC128 mice	Reviewed in Leoni and Caccia, 2015
Reduced cholesterol transporter genes (ABCA1, ABCG1, ABCG4, APOE) and MBP	Reduced ApoE-mediated cholesterol transport and supply from astrocytes to neurons Inefficient ApoE-dependent cholesterol removal from neurons Reduced lipidation of ApoE	Primary astrocytes from R6/2 HD and YAC128 mice	Reviewed in Leoni and Caccia, 2015
Decreased striatal cholesterol synthesis	Reduced SREBP2 translocation Probable impaired capability of mut HTT to upregulate LXR and LXR-targeted genes	R6/2 HD mice	Reviewed in Leoni and Caccia, 2015
Decrease mitochondrial membrane cholesterol levels	Increased mitochondrial membrane fluidity	BACHD rats	Reviewed in Leoni and Caccia, 2015
Increased mitochondrial conjugated dienes (2.5x), cholesterol (2X), and glycolipids (4.3x) in striatum Increased 9.5-fold mitochondrial cholesterol/phospholipid ratio	Oxidation of mitochondrial membrane lipids by ROS Probable increase of plasma cholesterol by disrupt of BBB Significant reduction of mitochondrial phospholipids in striatum Decreased mitochondrial membrane fluidity	Rats 3-NP treated	Mehrotra et al., 2015
	ALA and ALCAR restore mitochondrial lipid composition altered by 3-NP	Rats 3-NP treated with or without ALA and/or ALCAR	
Accumulation of cholesterol in lipid droplets, caveolae and lipid rafts	MutHTT-dependent impaired trafficking of post Golgi caveolin-1	Striatal neurons from Hdh (Q150/150)	Reviewed in Leoni and Caccia, 2015

*3-NP, 3-nitropropionic acid; 24S-OHC, 24-hydroxycholesteol; 27-OHC, 27-hydroxycholesterol; ALA, alpha-lipoic acid; ALCAR, acetyl-l-carnitine; BBB, blood-brain barrier; fdft1, farnesyl-diphosphate farnesyl transferase 1 gene; hmgcr, hydroxyl-methyl-glutaryil-CoA-reductase gene; HD, Huntington's disease; HMGCR, HMG CoA reductase; HMGS1, HMG CoA synthase; htt, huntingtin; LXR, liver X receptor; MBP, myelin basic protein; mut HTT, mutant huntingtin; ROS, reactive oxygen species; PGC1α, Peroxisome-proliferator-activated receptor gamma coactivator 1 alpha; Tg, transgenic; WT, wild-type*.

### MAM in cholesterol trafficking and neurodegeneration

As mentioned above MAM have emerged as specialized subdomains that play key roles in cholesterol and phospholipids metabolism, calcium signaling, apoptosis, ER stress, autophagy, and mitochondrial dynamics and integrity (Vance, 2014; van Vliet et al., 2014; Giorgi et al., 2015; Area-Gomez and Schon, 2016; van Vliet and Agostinis, 2017). Alterations in the signaling and/or components of MAMs are implicated in several neurodegenerative disorders including AD, PD, HD, amyotrophic lateral sclerosis (ALS), frontotemporal dementia (FTD), and Charcot-Marie-Tooth disease (CMT; Giorgi et al., 2015; Krols et al., 2016; Area-Gomez and Schon, 2017). MAM is a specialized ER-subdomain that connects mitochondria and the ER, both physically and biochemically (Giorgi et al., 2015; Area-Gomez and Schon, 2016). This is quite relevant for neurons, since their morphology and function involve inter-organelle communication via vesicular transport that depends on MAMs to exchange metabolites and signaling molecules. Importantly, it has been described that the ER portion of MAM is an intracellular lipid-raft domain given its detergent-resistance (Area-Gomez et al., 2012). Moreover, PSEN1/2 and γ-secretase are located predominantly at the MAM (Area-Gomez et al., 2012), in concordance with observations that localize Aβ within mitochondria in AD-patients and transgenic AD-mice (Lustbader et al., 2004; Caspersen et al., 2005), and in neuroblastoma cells (Hayashi et al., 2012; Zheng et al., 2013). However, although there has been recent evidence against the support for the amyloidogenic APP processing in mitochondria from human neuroblastoma cell lines based on an iodixanol centrifugation gradient that separated mitochondria from lysosomes (Mamada et al., 2017), this approach disrupted the structure of MAM, which precluded functional analyses of APP processing in these domains. In contrast to these findings, MAMs isolated by a Percoll gradient centrifugation from cellular models overexpressing wild type APP or APP harboring familial AD mutations and from brains of double-transgenic AD-mice model, revealed the presence of APP and its catabolites in conjunction with APP-processing active forms of both β- and γ-secretases (Del Prete et al., 2017). The impact APP processing in this subcellular localization was assessed by the overexpression of APP or BACE1 in human HEK293 cells (expressing low endogenous BACE1 expression) and N2a murine neuroblastoma cell line. APP overexpression (but not BACE1) reduced mitochondrial respiration and treatment with GSI to inhibit γ-secretase rescued this phenotype (Schaefer et al., 2016). Moreover, further analysis revealed that an impaired mitochondrial respiration is associated with both intracellular Aβ levels and an increased APP/Aβ-mitochondria apposition (Schaefer et al., 2016). In addition to Aβ-mediated mitochondrial toxicity, Del Prete and coworkers described that APP and Aβ accumulated at MAM interact with key MAM's proteins that controls mitochondria and ER functions and contributes to an increase in ER-mitochondria contact sites (Del Prete et al., 2017). In fact, cells containing PSEN1-mutant familial AD also showed MAM upregulation, which was mitigated by knocking down MFN2 (Area-Gomez et al., 2012), a MAM-resident protein involved in mitochondrial fusion and ER-mitochondrial connectivity. Moreover, PSEN1-mutant cells and AD fibroblasts exhibited a significant increase in the levels of both phosphatidylserine (synthesized at MAM) and phosphatidylethanolamine (converted at mitochondria; Area-Gomez et al., 2012), indicating an increase in MAM activity, in concordance with the altered phospholipid profiles seen in AD (Mielke et al., 2010).

Interestingly, amyloidogenic processing of pathogenic APP at MAM seems to promote lipid droplets accumulation, and effect that is reversed upon inhibition of both β- or γ-secretases (Del Prete et al., 2017). Indeed, this outcome of increased lipid droplets is consistent with the presence of higher activity of ACAT1, a MAM-resident protein, in both PSEN1-mutant cells and in cells from AD-patients compared with controls, correlating with increased lipid droplets (Area-Gomez et al., 2012). These findings are also consistent with the observation that ACAT1 activity is necessary for the production of Aβ (Puglielli et al., 2004; Murphy et al., 2013; Zhu et al., 2015). For instance, both human fibroblasts and explanted mouse neurons treated with astrocyte-conditioned medium containing ApoE4 show a significant increase of MAM activity measured by both MAM-mediated phospholipid transport and ACAT1-mediated cholesteryl ester synthesis and lipid droplet formation (Tambini et al., 2016), suggesting a link between upregulation in MAM function and ApoE4 as a risk factor of AD (Figure 4).

Recently, among most key components of the cholesterol/steroid biosynthesis and transport pathways mapped from highly purified MAM fractions from mouse liver using in-depth mass spectrometry characterization; CAV1 was identified as a specific integral component of MAMs (Sala-Vila et al., 2016). Using a cytometry-based FRET assay through a rapamycin-inducible fluorescent artificial ER-mitochondria tethering reporters on embryonic fibroblast from wild-type and CAV1-deficient mice, Salas-Vila et al. observed a reduction in FRET signal in the CAV1-deficient cells indicating decreased MAM extension/stability associated with CAV1 deficiency that was accompanied by a significant increase in free cholesterol accumulation at MAM (Sala-Vila et al., 2016). Moreover, quantitative mass spectrometry showed alterations in steroid biosynthesis (depletion of HMGC synthase at MAM) and inorganic ion transport (MAM-enriched membrane cation transporters) in CAV1-deficient mice (Sala-Vila et al., 2016). Importantly, the absence of CAV1 has been related with aging and AD-like neuropathology, since similar to older WT mice, young CAV1-deficient mice exhibit reduction of synapses and degeneration (Head et al., 2010). Moreover, hippocampus from young CAV1-deficient mice show increased AD-like markers (Aβ, P-tau, astrogliosis, and neurodegeneration) compared with young WT mice (Head et al., 2010).

In addition to the regulation of lipid droplets, MAMs play a key role in transferring stress signals from the ER to mitochondria emerging as a central site involved in the upregulation of ER chaperones during the early adaptive phases of ER stress and in the regulation of steroidognesis (van Vliet and Agostinis, 2017). For instance, GRP78 has been localized in MAM in rat testes and MA-10 cells where facilitates StAR intermediate state folding that governs the folding state required for its delivery to OMM to promote steroidogenesis (Prasad et al., 2017). Indeed, siRNA-dependent GRP78 knockdown prevents StAR intermediate state emergence leading to the degradation of the unfolded StAR, which translates in the deletion of functional StAR and hence shutdown of mitochondrial steroidogenesis (Prasad et al., 2017). Thus, based on these findings, it is conceivable that GRP78 acts as an acute regulator of mitochondrial cholesterol loading and steroidogenesis at the MAM by regulating StAR folding. This outcome may be of relevance as GRP78 levels correlate with Braak staging of patients with AD and increased expression of GRP78 in AD models constitutes an early molecular event in the Aβ-induced ER stress leading to mGSH depletion through mitochondrial cholesterol loading, causing neurotoxicity and neuroinflammation (Barbero-Camps et al., 2014; Placido et al., 2014).

Together, all these observations not only support the concept that MAMs emerge as intracellular sites for amyloidogenic APP processing, but also that MAM agglutinates several other biochemical and morphological features typically observed at early stages of AD (Figure 2B), especially mitochondrial dysfunction as a common feature in many neurodegenerative diseases, defining the so called MAM hypothesis as a key event for AD pathogenesis (Area-Gomez and Schon, 2017).

## Concluding remarks

Cholesterol is a vital component of membrane bilayers that plays both structural and functional roles, regulating a wide range of signaling pathways and cell functions. Given these key biological functions, it seems evident that cholesterol homeostasis dysregulations have been associated with the onset of hereditary brain disorders and major neurodegenerative diseases. However, in the case of AD, it is unclear whether increased or decreased brain cholesterol levels are involved in brain dysfunction and disease progression. These findings suggest that the distribution may be more important than the alterations in total cholesterol levels for neurodegeneration. Indeed, cholesterol trafficking and accumulation in PM, endolysosomes and mitochondria via MAM may be more relevant to disease progression by regulating specific signaling pathways and intracellular compartment function. This premise has been recently well-illustrated with the case of mitochondrial cholesterol accumulation in NPC disease and AD disease. Although, the pathogenesis of both diseases is markedly different, both exhibit a common feature of increased cholesterol accumulation, which exceeds its metabolism within mitochondria. The accumulation of cholesterol in mitochondrial membranes, particularly in IMM, impairs the transport of GSH from cytosol to the mitochondrial matrix, rendering mitochondria more susceptible to Aβ-induced ROS generation and neurotoxicity as well as in the modulation of kinase-dependent tau phosphorylation. The accumulation of mitochondrial cholesterol involved an ER stress-mitochondrial cross-talk resulting in the increased expression of StARD1-mediated trafficking of cholesterol to IMM. A better understanding of the functional consequences and mechanisms of mitochondrial or endolysosomal enrichment in cholesterol may provide further insight in the pathogenesis of major neurodegenerative diseases and in the design of novel and improved therapies for treatment. In this regard, the Table 4 resume the latest approaches that therapeutically targeted several factors involved in the brain's cholesterol homeostasis regarding reduction/amelioration of neurodegeneration in animal models for AD. Among these approaches, improving mitochondrial antioxidant defense in a specific fashion may stand as a novel approach of relevance for neurodegeneration, such as AD, and lysosomal disorders like NPC. In this regard, the boost of cytosolic GSH levels may be insufficient to guarantee mitochondrial GSH levels due to the restriction of increased mitochondrial cholesterol accumulation, which is bypassed by GSH permeable prodrugs that diffuse into mitochondria. The combination of this approach with other chemically modified scavengers targeted to mitochondria may be a promising approach for the treatment of neurodegenerative diseases.

**Table 4:** Selected latest brain cholesterol homeostasis factors therapeutically targeted in Alzheimer disease.

**Factor targeted**	**Effects observed**	**Treatment (via of administration)**	**Experimental model**	**References**
mGSH impairment	Restored mGSH Reduced protein carbonyl content in brain Partial prevention the activation of tau kinases Reduced tau aggregation Reduced hippocampal Aβ42 deposition Prevention of synaptic degeneration	GSH ethyl ester (i.p.)	APP/PS1 AD mice overexpressing SREBP2	Barbero-Camps et al., 2013
Mitochondrial cholesterol accumulation	Reduced hippocampal ER stress Reduced apoptosis in the hippocampus Downregulation of SREBP2 expression Prevention of mitochondrial cholesterol loading Prevention of mGSH depletion Protection against Aβ-induced neurotoxicity	4-phenylbutyric acid (i.p.)	APP/PS1 AD mice	Barbero-Camps et al., 2014
Cholesterol transport	Improved spatial learning performance Restored resting-state functional connectivity Reduced brain Aβ plaque load	Monoclonal anti-ApoE antibody (i.p.)	APP/PS1 AD mice	Liao et al., 2014
ACAT1/2	Reduced brain Aβ deposition Rescued the cognitive deficits	CP-113,818 (i.p.) Avasimibe (i.p.)	hAPP751 mice	Reviewed in Shibuya et al., 2015
ABCA1	Reversal of the ApoE4-driven neurodegeneration and cognitive deficits	Bexarotene (oral gavage) or CS-6253 (i.p.)	ApoE4-TR mice	Boehm-Cagan and Michaelson, 2014; Boehm-Cagan et al., 2016b
Cholesterol transport	Reduced neuroimmflamation	Recombinant ApoA-I-Milano (i.v.)	APP23 Tg mice	Fernandez-de Retana et al., 2017
LE/LY cholesterol content	Decreased lysosonal Aβ accumulation Increased lysosomal proteolytic activity and autophagy	CYCLO (icv)	TgCRND8 mice	Yang et al., 2017a
HMGCR	Facilitated clearance of Aβ across BBB Increased levels of LRP1/2 and apoJ Increased secreted Aβ oligomers and reduced Aβ uptake	simvastatin (oral gavage)	3x Tg AD	Zandl-Lang et al., 2017
CYP46A1	Reduction of amyloid burden and neuroimmflamation Improved long-term spatial memory Increased supervivency	Efavirenz (drinking water)	5X FAD mice	Mast et al., 2017

*Avisamibe and CP-113,818, ACAT1/2 inhibitors; Bexarotene and CS-6253, RXR agonist; CYCLO, 2-hydroxypropyl-β-cyclodextrin; icv, intracerebroventricular; Efavirenz, anti-HIV drug; mGSH, mitochondrial glutathione*.

## Author contributions

FA, CG-R, and JF-C. discussed findings, analyzed literature, and wrote the manuscript.

### Conflict of interest statement

The authors declare that the research was conducted in the absence of any commercial or financial relationships that could be construed as a potential conflict of interest.

## Abbreviations

ABC: ATP-binding cassette transporters
ABCA: ABC subfamily A transporters
Aβ: amyloid-β peptide
ACAT1: acylcoenzyme A:cholesterol acyltransferase 1
AD: Alzheimer disease
ANT: adenine nucleotide transporter
ApoA-I: apolipoprotein A-I
ApoE: apolipoprotein E
ApoER2: ApoE receptor 2
APP: amyloid precursor protein
APP/PS1/SREBP2: APP/PS1 mice overexpressing SREBP2
ATAD3: AAA domain-containing protein 3
BACE1: Beta-secretase 1
BBB: blood brain barrier
CAV: caveolins
CatD: cathepsin D
CNS: central nervous system
CSF: cerebrospinal fluid
ER: endoplasmic reticulum
HD: Huntington disease
HDL: high-density lipoproteins
HMG-CoA: 3-hydroxy-3-methylglutaryl-CoA
HMGR: HMG-CoA reductase
IMM: inner mitochondrial membrane
LDLR: low-density lipoprotein receptor
LE/LY: late endosome/lysosome
LRP-1: lipoprotein receptor-related protein 1
LRO: lysosome-related organelle
LXR: nuclear liver X receptor
MAM: mitochondria associated ER membranes
MCS: membrane contact sites
MS: multiple sclerosis
mtDNA: mitochondrial DNA
mGSH: mitochondrial GSH
NPC1: Niemann-Pick type C1 protein
NPC2: Niemann-Pick type C2 protein
OGC: 2-oxoglutarate carrier
OSBP: oxysterol-binding protein
ORPs: OSBP-related proteins
OMM: outer mitochondrial membrane
PD: Parkinson disease
PM: plasma membrane
PI4P: phosphatidylinositol 4-phosphate
PSEN1/2: presenilins ½
ROS: reactive oxygen species
SCAP: SREBP cleavage activating protein
SREBP2: sterol regulatory element protein 2
StAR: steroidogenic acute regulatory protein
START: StAR-related domain lipid transfer
TSPO: Translocator protein
VDAC: voltage-dependent anion channel
VLDL: very low-density lipoproteins
MPP+: 4-methylpyridinium
24-OHC: 24-hydroxycholesterol
24-DHCR: 24-dehydrocholesterol reductase
7-DHCR: 7-dehydrocholesterol reductase.
